# Associations of Workplace Bullying and Harassment with Pain

**DOI:** 10.3390/ijerph10104560

**Published:** 2013-09-25

**Authors:** Jiro Takaki, Toshiyo Taniguchi, Kumi Hirokawa

**Affiliations:** 1 Department of Public Health and Occupational Medicine, Mie University Graduate School of Medicine, 2-174 Edobashi, Tsu, Mie 514-8507, Japan; 2 Department of Welfare System and Health Science, Okayama Prefectural University, 111 Kuboki Soja, Okayama 719-1197, Japan; E-Mail: taniguti@fhw.oka-pu.ac.jp; 3 Department of Nursing, Baika Women’s University, 2-19-5 Shukunosho, Ibaraki, Osaka 567-8578, Japan; E-Mail: k-umi@umin.ac.jp

**Keywords:** workplace bullying, harassment, depression, pain, prevalence ratio

## Abstract

The aim of this study was to investigate associations of workplace bullying and harassment with headache, stiffness of the neck or shoulders, lumbago, and pain of two or more joints. The subjects in this cross-sectional study were recruited from workers (*n =* 1,913) at 35 healthcare or welfare facilities in Japan. Because of non-participation or missing data, the number of subjects included in the analysis varied (response rate ≥ 77.1%). Workplace bullying and harassment were assessed using the Negative Acts Questionnaire. Depression was assessed using the Brief Job Stress Questionnaire. The frequency of pain experienced by workers in the previous month was evaluated using a four-point scale. Many of the associations of person-related bullying, work-related bullying, and sexual harassment with headache, stiffness of the neck or shoulders, lumbago, and pain of two or more joints were positive and significant (*p* < 0.05). Even after adjustment for depression, some of the associations remained significant (*p* < 0.05). For example, changes in the prevalence ratio for headache associated with a 1-point increase in the work-related bullying score were 1.05 (95% confidence interval (CI) 1.01 to 1.09) in men and 1.03 (95% CI 1.01 to 1.05) in women after adjustment for age, marital status, employment status, work shift, and depression.

## 1. Introduction

According to the International Labour Office (ILO), the International Council of Nurses (ICN), the World Health Organization (WHO), and the Public Services International (PSI), bullying (or mobbing) is “repeated and over time offensive behaviour through vindictive, cruel or malicious attempts to humiliate or undermine an individual or groups of employees,” and harassment is “any conduct based on age, disability, HIV status, domestic circumstances, sex, sexual orientation, gender reassignment, race, colour, language, religion, political, trade union or other opinion or belief, national or social origin, association with a minority, property, birth or other status that is unreciprocated or unwanted and which affects the dignity of men and women at work” [1]. Many victims of these actions demonstrate depressive or somatic symptoms [1].

In a recent cross-sectional study, neck pain was associated with intimidation at work and with unwanted sexual attention in bivariate analyses in both genders [2]. In another recent study, new onset chronic neck pain during follow-up was associated with current workplace bullying, earlier bullying at the present workplace, and earlier bullying in another workplace in women, but not men [3]. However, to the best of our knowledge, the associations of workplace bullying and harassment with headache, stiffness of the neck or shoulders, lumbago, and pain of two or more joints have not yet been investigated. We hypothesized that workplace bullying and harassment are associated with headache, stiffness of the neck or shoulders, lumbago, and pain of two or more joints. Because a previous study reported gender difference [3], we analyzed the data in men and women separately.

## 2. Methods

### 2.1. Subjects

The subjects in this study were recruited from workers (*n =* 1,931) at 35 healthcare or welfare facilities in Japan. Questionnaires were mailed to the organizations and distributed to the workers. The purpose and procedure of the survey were explained to the participants in the documents. Written informed consent was obtained from all participants, who were not compensated for their participation. A total of 1,642 questionnaires were returned in sealed envelopes (response rate, 85.0%). Because of missing data, the number of subjects included in the analysis varied (Table 1, Table 2, Table 3 and Table 4). This study was approved by the ethics committee of the Okayama Prefectural University.

### 2.2. Measures

From August to September, 2009, participants completed a self-administered questionnaire including background information such as age, gender, marital status, employment status, and work shift as well as measures of workplace bullying and harassment, depression, and pain.

Workplace bullying and harassment were assessed using the Negative Acts Questionnaire (NAQ) [4]. The NAQ is a self-administered questionnaire originally developed by Einarsen and Raknes that measures exposure to specific negative acts typical of bullying [4]. It contains items that refer to both direct and indirect behaviors, but it does not require respondents to label themselves as targets of bullying. Respondents indicated on a five-point scale (1 = never, 2 = now and then, 3 = monthly, 4 = weekly, 5 = daily) whether they have experienced the designated negative acts in the context of their job [4]. The NAQ was translated into Japanese using a back-translation method, and the internal consistency reliability and factor and construct validity are reportedly acceptable [1]. A cross-validation study revealed three subscales of the NAQ: person-related bullying (six items such as “Gossip or rumors about you”; score range, 6–30), work-related bullying (three items such as “Someone withholding necessary information so that your work gets complicated”; score range, 3–15), and sexual harassment (three items such as “Unwanted sexual advances”; score range, 3–15) [1].

The frequency of pain (headache, stiffness of the neck or shoulders, lumbago, and pain of two or more joints) experienced by workers in the previous month was evaluated using a four-point scale (1 = almost never, 2 = sometimes, 3 = often, 4 = very often). Participants who answered “2,” “3,” or “4” for a particular symptom were categorized as having that symptom; participants who answered “1” for a particular symptom were categorized as not having that symptom.

Depression was evaluated using a self-reported Brief Job Stress Questionnaire (BJSQ) published in a research report on stress in the workplace and its impact on workers’ health [5]. The BJSQ was developed in Japan with the support of the Japanese Ministry of Labour and has been widely used in Japan to evaluate work-related stressful situations in various clinical and occupational settings [6,7,8,9,10]. In the BJSQ, six items, such as “I feel sad,” are used to measure workers’ depression. The response option is based on the frequency in the previous month and is scored using a four-point scale (1 = almost never, 2 = sometimes, 3 = often, 4 = very often). We calculated a total score (range, 6–24) for the six items as a measure of depression. Cronbach’s α for the depression scale has been reported as 0.86 [5] and was 0.89 in the present study.

### 2.3. Statistical Analyses

Variables of age and depression were compared using the unpaired *t*-test, variables of workplace bullying and harassment were compared using the Mann-Whitney U test, and categorical variables were compared using the chi-square test. SPSS version 20.0 were used for these tests. Based on the recommendation of Barros and Hirakata, prevalence ratios (PRs) and their 95% confidence intervals (CIs) were calculated by Cox regression with constant time at risk and robust variance using Stata/SE 10 [11]. All tests were two-tailed, and statistical significance was set at *p* < 0.05.

## 3. Results

Participant characteristics according to gender are shown in Table 1. On average, men were significantly younger than women. Scores for person-related bullying (mean score, 8.3 for men *versus* 7.8 for women) and sexual harassment (mean score, 3.38 for men *versus* 3.25 for women) were significantly higher in men than in women.

**Table 1:** Participant characteristics according to gender.

		Men ( *n =* 367) ^a^			Women ( *n =* 1,266) ^a^			*p* ^b^	Number of missing data
		Mean	SD	Range	Mean	SD	Range		
Age (years)		31.9	9.7	18–74	39.2	12.8	17–73	<0.001	5
Depression		11.2	4.2	6–24	10.9	4.1	6–24	0.358	36
Person-related bullying		8.3	3.3	6–29	7.8	2.9	6–30	0.036	83
Work-related bullying		5.1	2.1	3–15	4.8	1.8	3–14	0.187	51
Sexual harassment		3.38	0.99	3–11	3.25	0.81	3–13	0.008	36
		n	%		n	%			
Marital status								<0.001	10
	Unmarried	186	51.0		444	35.3			
	Married	169	46.3		629	50.0			
	Divorced or widowed	10	2.7		185	14.7			
Employment status								<0.001	13
	Regular	345	94.3		975	77.8			
	Contractual	21	5.7		279	22.2			
Work shift								<0.001	12
	Shift work without night shift	30	8.2		207	16.5			
	Shift work with night shift	251	68.8		566	45.1			
	Regular daytime work	84	23.0		483	38.5			
Type of occupation								Not applicable	45
	Professional caregiver	290	81.0		879	71.5			
	Nurse	4	1.1		158	12.8			
	Clerk	27	7.5		45	3.7			
	Nutritionist	0	0.0		75	6.1			
	Others	37	10.4		73	5.9			
Having headache		190	51.9		876	69.7		<0.001	9
Having stiffness of neck or shoulders		247	67.9		1047	83.3		<0.001	10
Having lumbago		247	67.9		983	78.2		<0.001	12
Having pain of two or more joints		145	39.6		671	53.3		<0.001	12

SD = standard deviation; ^a^ N of missing data for gender was 9; ^b^ Variables of age and depression were compared using the unpaired *t*-test, variables of workplace bullying and harassment were compared using the Mann-Whitney U test, and categorical variables were compared using the chi-square test.

**Table 2:** Changes in the prevalence ratio associated with a 1-point increase in the person-related bullying score.

		Men			Women		
		PR	95% CI	N ^a^	PR	95% CI	N ^a^
*Headache*							
	Crude	**1.04**	**(1.01, 1.07)**	347	**1.03 **	**(1.02, 1.04)**	1,196
	Adjusted for demographics ^b^	**1.04**	**(1.01, 1.07)**	343	**1.03 **	**(1.02, 1.04)**	1,169
	Additionally adjusted for depression	1.01	(0.98, 1.04)	343	**1.01 **	**(1.00, 1.02)**	1,145
*Stiffness of neck or shoulders*							
	Crude	1.02	(0.99, 1.04)	346	1.01	(1.00, 1.01)	1,197
	Adjusted for demographics ^b^	1.02	(0.99, 1.04)	342	1.01	(1.00, 1.01)	1,170
	Additionally adjusted for depression	1.00	(0.97, 1.02)	342	1.00	(0.99, 1.01)	1,146
*Lumbago*							
	Crude	1.01	(0.99, 1.04)	346	**1.02 **	**(1.02, 1.03)**	1,196
	Adjusted for demographics ^b^	1.02	(0.99, 1.04)	342	**1.02 **	**(1.02, 1.03)**	1,169
	Additionally adjusted for depression	1.00	(0.98, 1.03)	342	**1.01 **	**(1.00, 1.02)**	1,146
*Pain of two or more joints*							
	Crude	**1.05 **	**(1.02, 1.09)**	347	**1.04 **	**(1.02, 1.05)**	1,197
	Adjusted for demographics ^b^	**1.06 **	**(1.02, 1.09)**	343	**1.04 **	**(1.03, 1.05)**	1,170
	Additionally adjusted for depression	1.03	(1.00, 1.06)	343	1.01	(0.99, 1.02)	1,147

PR = prevalence ratio, CI = confidence interval; ^a^ N may vary due to missing data; ^b^ Age, marital status, employment status, and work shift; Bold values signify statistical significance.

**Table 3:** Changes in the prevalence ratio associated with a 1-point increase in the work-related bullying score.

		Men			Women		
		PR	95% CI	N ^a^	PR	95% CI	N ^a^
*Headache*							
	Crude	**1.09**	**(1.05, 1.13)**	355	**1.06 **	**(1.04, 1.08)**	1,220
	Adjusted for demographics ^b^	**1.09**	**(1.06, 1.13)**	352	**1.06 **	**(1.04, 1.08)**	1,193
	Additionally adjusted for depression	**1.05**	**(1.01, 1.09)**	349	**1.03 **	**(1.01, 1.05)**	1,168
*Stiffness of neck or shoulders*							
	Crude	**1.04 **	**(1.01, 1.07)**	353	**1.02 **	**(1.01, 1.04)**	1,220
	Adjusted for demographics ^b^	**1.04 **	**(1.01, 1.07)**	350	**1.02 **	**(1.01, 1.03)**	1,193
	Additionally adjusted for depression	1.01	(0.98, 1.04)	347	1.01	(0.99, 1.02)	1,168
*Lumbago*							
	Crude	1.01	(0.98, 1.05)	353	**1.03 **	**(1.02, 1.05)**	1,220
	Adjusted for demographics ^b^	1.02	(0.99, 1.05)	350	**1.04 **	**(1.02, 1.05)**	1,193
	Additionally adjusted for depression	0.99	(0.96, 1.03)	347	**1.02 **	**(1.00, 1.03)**	1,169
*Pain of two or more joints*							
	Crude	**1.08 **	**(1.03, 1.13)**	355	**1.07 **	**(1.04, 1.09)**	1,221
	Adjusted for demographics ^b^	**1.10 **	**(1.04, 1.15)**	352	**1.07 **	**(1.04, 1.09)**	1,194
	Additionally adjusted for depression	1.04	(0.99, 1.10)	349	1.01	(0.99, 1.04)	1,170

PR = prevalence ratio, CI = confidence interval; ^a^ N may vary due to missing data; ^b^ Age, marital status, employment status, and work shift; Bold values signify statistical significance.

**Table 4:** Changes in the prevalence ratio associated with a 1-point increase in the sexual harassment score.

		Men			Women		
		PR	95% CI	N ^a^	PR	95% CI	N ^a^
*Headache*							
	Crude	1.08	(0.99, 1.18)	361	**1.08 **	**(1.05, 1.10)**	1,229
	Adjusted for demographics ^b^	1.08	(0.99, 1.19)	357	**1.07 **	**(1.04, 1.10)**	1,201
	Additionally adjusted for depression	1.03	(0.94, 1.12)	354	1.03	(1.00, 1.06)	1,176
*Stiffness of neck or shoulders*							
	Crude	1.01	(0.94, 1.08)	359	**1.03 **	**(1.01, 1.05)**	1,229
	Adjusted for demographics ^b^	1.01	(0.94, 1.09)	355	**1.02 **	**(1.00, 1.05)**	1,201
	Additionally adjusted for depression	0.97	(0.90, 1.04)	352	1.02	(1.00, 1.04)	1,176
*Lumbago*							
	Crude	**1.07 **	**(1.01, 1.15)**	359	**1.05 **	**(1.03, 1.07)**	1,229
	Adjusted for demographics ^b^	**1.07 **	**(1.00, 1.14)**	355	**1.05 **	**(1.03, 1.07)**	1,201
	Additionally adjusted for depression	1.04	(0.98, 1.11)	352	**1.03 **	**(1.00, 1.05)**	1,177
*Pain of two or more joints*							
	Crude	**1.13 **	**(1.03, 1.24)**	361	**1.09 **	**(1.06, 1.13)**	1,230
	Adjusted for demographics ^b^	**1.13 **	**(1.02, 1.25)**	357	**1.12 **	**(1.08, 1.16)**	1,202
	Additionally adjusted for depression	1.06	(0.96, 1.17)	354	**1.06 **	**(1.02, 1.10)**	1,178

PR = prevalence ratio, CI = confidence interval; ^a^ N may vary due to missing data; ^b^ Age, marital status, employment status, and work shift; Bold values signify statistical significance.

Marital status, employment status, and work shift were significantly different between men and women. Significantly more women than men experienced each type of pain. Changes in the PR associated with a 1-point increase in the person-related bullying score are shown in Table 2. Headache was significantly positively associated with person-related bullying bivariately and after adjustment for demographics in both genders, and after adjustment for demographics and depression only in women. Stiffness of neck or shoulders was not significantly associated with person-related bullying. Lumbago was significantly positively associated with person-related bullying bivariately, after adjustment for demographics, and after adjustment for demographics and depression in women, but not in men. Pain of two or more joints was significantly positively associated with person-related bullying bivariately and after adjustment for demographics in both genders.

Changes in the PR associated with a 1-point increase in the work-related bullying score are shown in Table 3. Headache was significantly positively associated with work-related bullying bivariately, after adjustment for demographics, and after adjustment for demographics and depression in both genders. Stiffness of neck or shoulders was significantly positively associated with work-related bullying bivariately and after adjustment for demographics in both genders. Lumbago was significantly positively associated with work-related bullying bivariately, after adjustment for demographics, and after adjustment for demographics and depression in women, but not in men. Pain of two or more joints was significantly positively associated with work-related bullying bivariately and after adjustment for demographics in both genders.

Changes in the PR associated with a 1-point increase in the sexual harassment score are shown in Table 4. Headache and stiffness of neck or shoulders were significantly positively associated with sexual harassment bivariately and after adjustment for demographics only in women. Lumbago and pain of two or more joints were significantly positively associated with sexual harassment bivariately and after adjustment for demographics in both genders, and after adjustment for demographics and depression only in women.

## 4. Discussion

Many of the associations of person-related bullying, work-related bullying, and sexual harassment with pain were positive and significant. The point estimates of the PRs in men and women were similar, and their 95% CIs in men and women overlapped each other. We found significant associations more frequently in women than in men probably because of relatively large sample sizes in women. After adjustment for depression, the degrees of the associations of workplace bullying and harassment with pain decreased, but some of the associations remained significant.

Workplace bulling and harassment can cause depression [2,12,13]. Patients with depression are likely to report somatic symptoms [14]. Nakao and Yano reported an association between depression and somatic symptoms in Japanese workers [15]. Psychological distress has been reported to be a strong risk factor for neck pain in several studies [2]. Thus, depression might be an intermediate factor, rather than a confounding factor, in the association of workplace bullying and harassment with pain [16]. By adjustment for a possible intermediate depression, a direct effect of workplace bulling and harassment on pain, an effect of workplace bulling and harassment on pain that is not mediated by depression, could be calculated [16]. Depression can also be affected by pain [2]. If depression was affected by pain, we could not regard depression as a confounding factor either, interpretation of adjustment for depression should be made with caution, and adjustment for depression could underestimate the true associations of workplace bullying and harassment with pain [16].

Even after adjustment for depression, some of the associations of workplace bullying and harassment with pain remained significant. Mechanisms other than just depression appear to play a role in the relationship of workplace bullying and harassment with pain. For example, the relationship between corticosteroids and stress is well known. Recent scientific and clinical evidence has demonstrated the direct role that steroids play in the generation of chronic pain [17]. Stress reactions caused by bullying or harassment may affect health by direct biological effects, prolonged physiological activation and lack of restitution, or compromised lifestyle and health behaviors [18].

The strength of this study was that we used a relatively large sample of workers to obtain reliable results. However, we must also note several limitations. First, we need to be cautious in interpreting causality in our results because the study used a cross-sectional design. Second, because we used convenience sampling, the results may not be applicable to the entire workforce. Many of the participants were professional caregivers and working with night shift. However, because we included workers from 35 various facilities and obtained a response rate of at least 77.1%, some generalizability can be expected. For example, more women than men experienced each type of pain. This is consistent with previous studies on neck pain [2,13]. Third, the observed variables were self-reported. More objective measurements are needed in future studies.

The present study supports the association of workplace bullying and harassment with pain. If the causality from workplace bullying and harassment to pain was confirmed, to help prevent pain in workers, measures to prevent workplace bullying or harassment should be considered. Prevention may include the introduction of occupational guidelines against bullying and harassment, active monitoring of specific workplace bullying or harassment, and taking action to deal with bullying using criminal, civil, social, or occupational laws [19]. Other measures may also be necessary, such as improving workplace social support, which can reduce workplace bullying [1,20,21].

## 5. Conclusions

Many of the associations of person-related bullying, work-related bullying, and sexual harassment with headache, stiffness of the neck or shoulders, lumbago, and pain of two or more joints were positive and significant. Even after adjustment for depression, some of the associations remained significant.
